# Passive leg raising and compression stockings: a note of caution

**DOI:** 10.1186/s13054-015-0955-0

**Published:** 2015-06-01

**Authors:** Cyril Jacob Chacko, Matt P Wise, Paul J Frost

**Affiliations:** Critical Care Unit, University Hospital of Wales, Heath Park, Cardiff, CF14 4XW Wales, UK

In a recent editorial in *Critical Care*, Monnet and Teboul emphasize the value of passive leg raising (PLR) as a reliable bedside indicator of fluid responsiveness in critically ill patients [1]. This is because PLR induces changes in venous return, regardless of the mode of ventilation or the underlying cardiac arrhythmias. In their eloquently written editorial, they provide five practical rules for performing a PLR maneuver.

The authors state that during PLR about 300 mL of blood is auto-transfused from the lower limbs and splanchnic circulation into the central compartment, resulting in an increased cardiac output. This maneuver has been validated in cohorts of patients with either hypovolaemic or septic shock [2, 3]. It is a prerequisite of the test that the volume of blood that is auto-transfused be sufficient to increase mean circulatory pressure which drives venous return [3]. We hypothesize that this may not be the situation in all patients and that, if there is no change in cardiac output, blood pressure, or right atrial pressure [4], an inadequate volume of blood will be returned to the right heart. This may be relevant in patients with elastic compression stockings, which are widely used to prevent venous thromboembolism in critically ill patients. The effectiveness of PLR as a measure of fluid responsiveness can be compromised by elastic compression stockings [5]. Consequently, we would recommend that elastic compression stockings be routinely removed prior to the PLR maneuver.

## Abbreviation

PLR: Passive leg raising

## References

[1] Monnet X, Teboul JL (2015). Passive leg raising: five rules, not a drop of fluid!. Crit Care.

[2] Xu Q, Yan J, Cai G, Chen J, Li L, Hu C (2014). Effect of two volume responsiveness evaluation methods on fluid resuscitation and prognosis in septic shock patients. Chin Med J (Engl).

[3] Monnet X, Teboul JL (2008). Passive leg raising. Intensive Care Med.

[4] Gupta K, Sondergaard S, Parkin G, Leaning M, Aneman A (2015). Applying mean systemic filling pressure to assess the response to fluid boluses in cardiac post-surgical patients. Intensive Care Med.

[5] Zogheib E, Defouilloy C, Mahjoub Y, Cherradi N, Moubarak M, Beloucif S (2007). Modification of hemodynamic effect after passive leg raising test by the use of elastic compression stocking [Abstract]. Intensive Care Med.

